# Leclercia Adecarboxylata Causing Necrotizing Fasciitis in an Immunocompetent Athlete Injecting Illicit Testosterone Supplements

**DOI:** 10.7759/cureus.11196

**Published:** 2020-10-27

**Authors:** Milan Kaushik, Aayush Mittal, Kathleen Tirador, Hanan Ibrahim, Sean Drake

**Affiliations:** 1 Internal Medicine, Wayne State University School of Medicine, Detroit, USA; 2 Internal Medicine, Henry Ford Health System, Detroit, USA

**Keywords:** leclercia adecarboxylata, necrotizing fasciitis, bacterial infection, herbal supplements, testosterone, illicit drugs, immunocompetent, rare pathogen, injection drug use, soft tissue infection

## Abstract

Leclercia adecarboxylata (L. adecarboxylata) is an uncommon and often misdiagnosed cause of multiple infection types including skin and soft tissue, cholecystitis, and septicemia. It commonly afflicts immunocompromised hosts or individuals who experience trauma in aquatic environments. We present a case where this bacteria causes necrotizing fasciitis as a consequence of injecting street bought testosterone supplements. This patient was treated successfully with excisional debridement of the wound as well as a one week course of Linezolid and Bactrim.

## Introduction

Leclercia adecarboxylata (L. adecarboxylata) is a rare, opportunistic gram-negative rod generally associated with cutaneous infections following aquatic exposures [1]. Here, we describe a case of an immunocompetent female who injected herself with an illicit steroid herbal combination for muscle growth. To our knowledge, this is the first case report of the emerging L. adecarboxylata causing necrotizing fasciitis in the setting of intramuscular injection in an immunocompetent host.

## Case presentation

**Figure 1:**
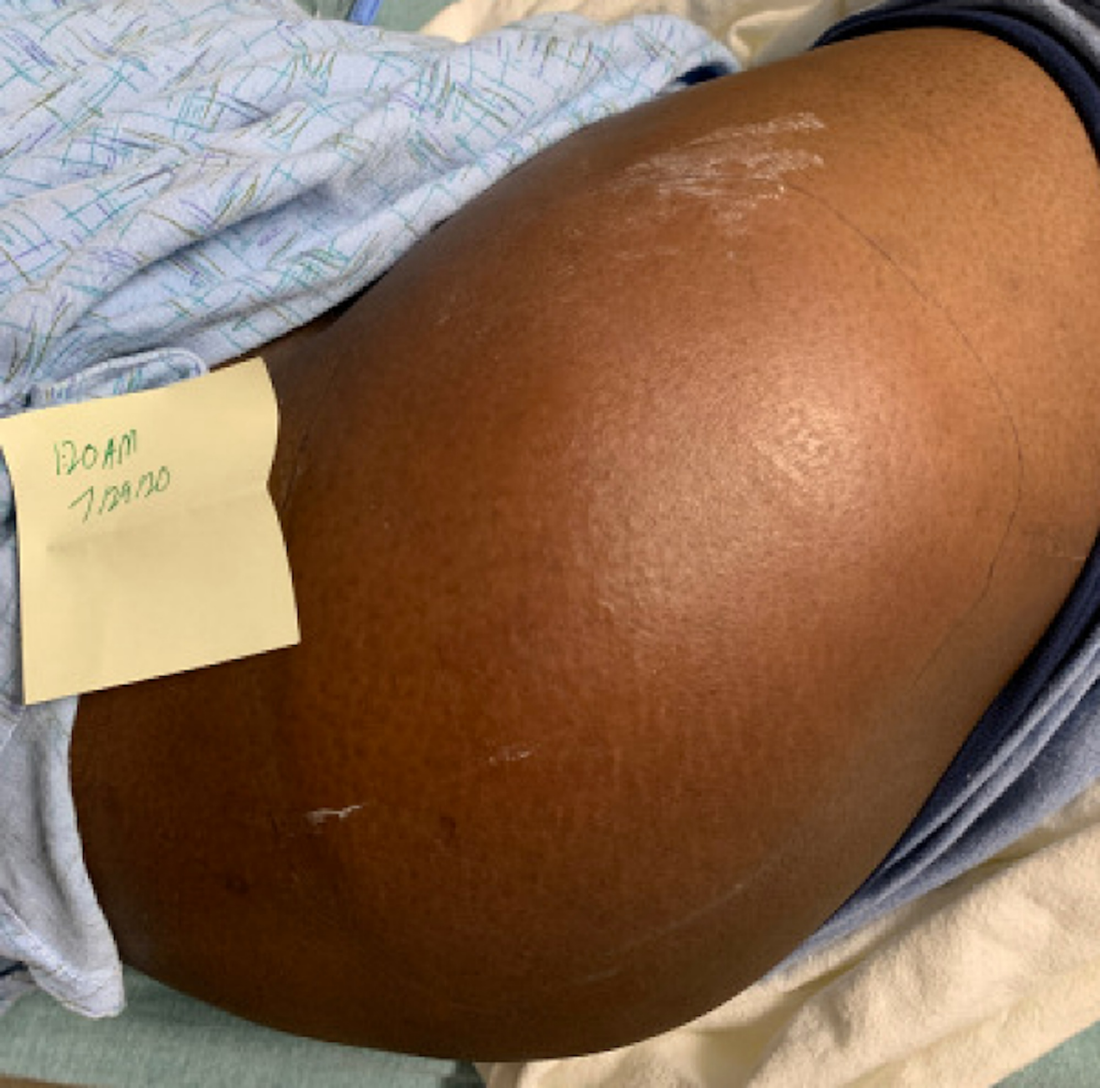
Patient's right hip on admission

**Figure 2:**
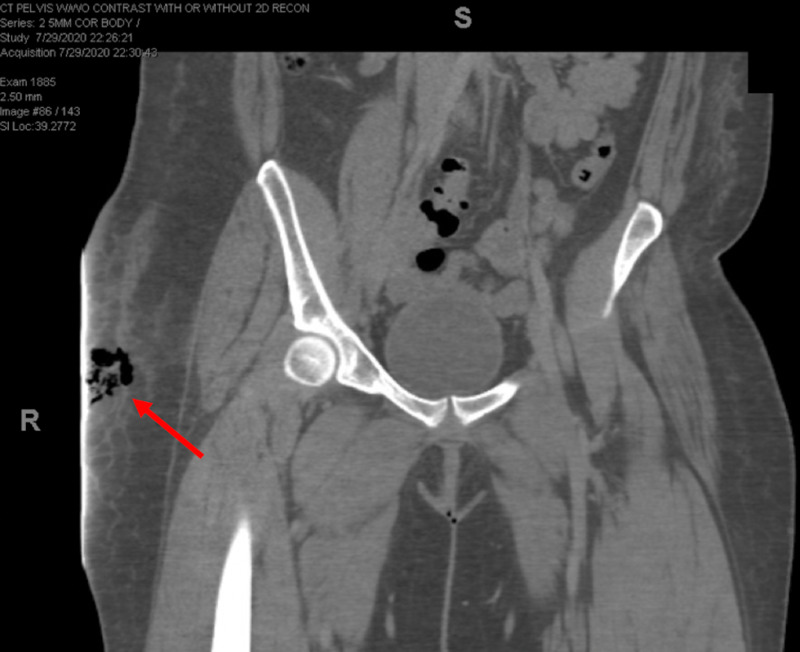
CT Pelvis demonstrating free gas in the patient's right hip soft tissue

**Figure 3:**
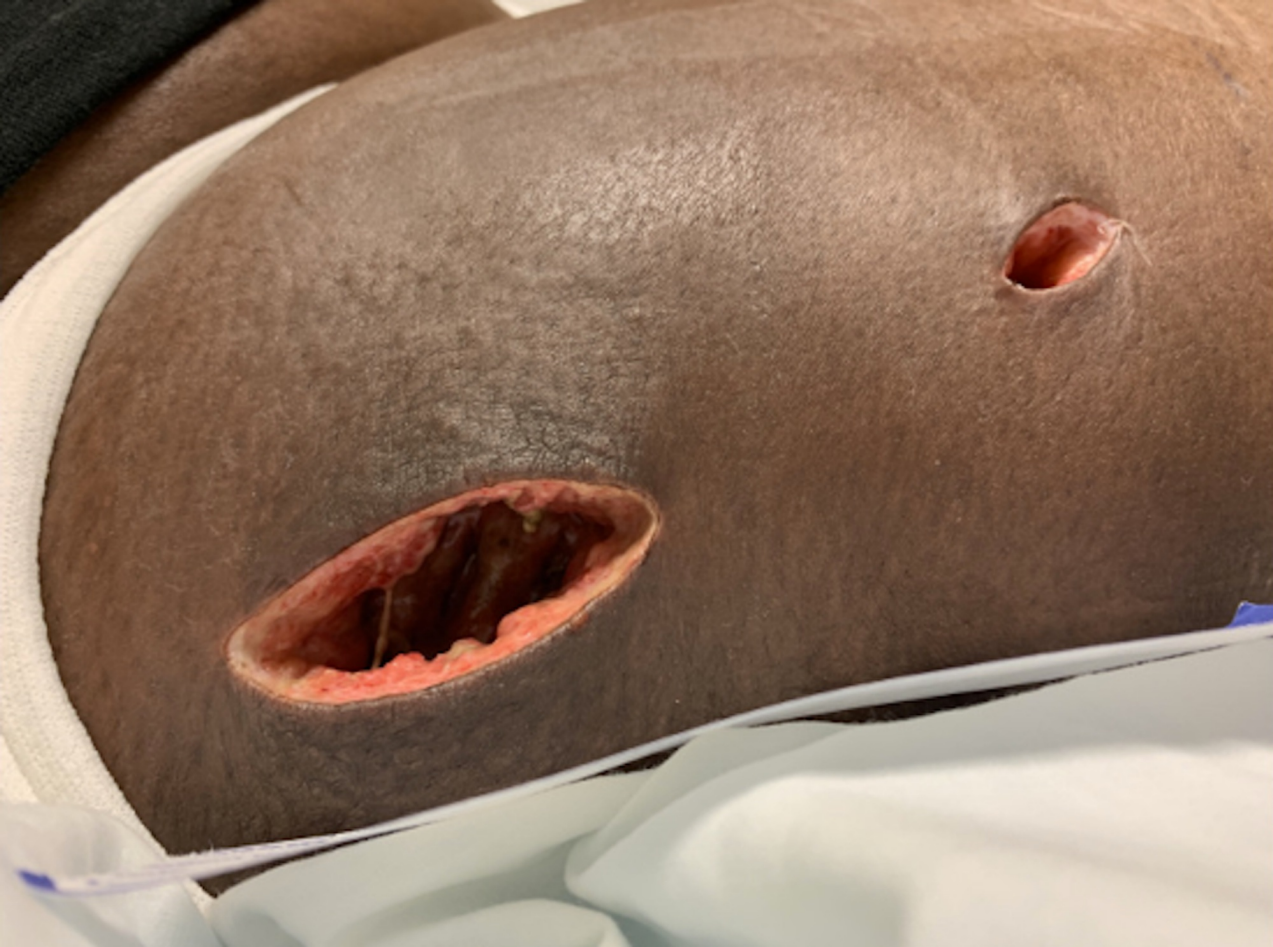
Postoperative incisions

A 22-year-old female was admitted to the hospital due to significant right hip pain. As an aspiring boxer, she had been injecting herself with a combination of testosterone and herbal supplements over the last six months to increase muscle mass. The patient states the drug combination and needles were both obtained from a street vendor. Two weeks prior to presentation, she developed redness surrounding the injection site and right hip pain which impaired her ambulation. She has a history of seizures on keppra, asthma, and schizoaffective disorder which required prior psychiatric hospital admission. She smokes five cigarettes daily and marijuana occasionally but denies any other drug use. In the emergency department, she had a temperature of 37.9 C, heart rate of 119, and normal blood pressure with labs notable for elevated C- reactive protein (CRP) of 19.5 mg/dL and erythrocyte sedimentation rate (ESR) of 79 mm/Hr but a normal white blood cell count. Her hip is demonstrated in Figure 1. Blood cultures were obtained and showed no growth. Preliminary ultrasound revealed edema and trace fluid in the right hip joint but CT Pelvis highlighted a possible uncontained gas-forming infection in the soft tissue, but no evidence of myositis, osseous infections, or hip joint involvement (Figure 2). After one day of treatment with Vancomycin and Zosyn, excisional debridement of the right hip subcutaneous tissue was performed (Figure 3). Pockets of gas, pus, and necrotic tissue were found down to the fascial level which were sent for culture. Rapid speciation identified many Leclercia adecarboxylata only resistant to ampicillin, Klebsiella pneumoniae, and Enterococcus casseliflavus. The patient was started on one week of double-strength Bactrim per os (PO) and one week of PO Linezolid 600 mg BID out of concern for vancomycin resistant enterococcus. The patient was monitored for five days following the procedure and was lost to follow up.

## Discussion

First discovered in 1962 as “Escherichia Adecarboxylata (E.coli),” this pathogen was described as a motile, gram-negative, oxidase-negative bacterium that shared many similar biochemical properties with Escherichia Coli [2]. Due to advancement in microbiology identification capabilities, there have been an increasing number of L. adecarboxylata infections diagnosed, primarily as an opportunistic pathogen. It has been hypothesized that certain cases of possible L. adecarboxylata infections may have previously been misdiagnosed as E. coli due to a lack of awareness surrounding the bacterium [3].

As we continue to advance our microbiology diagnostic abilities, L. adecarboxylata is of growing importance in clinical literature which indicates that infections have manifested as septicemia [4], peritonitis [5], posttraumatic polymicrobial wound/soft tissue infection [6], and cholecystitis [7]. Although typically seen as an opportunistic infection in immunocompromised individuals, there have been increasing reports of L. adecarboxylata present in immunocompetent hosts who suffer from trauma in aquatic environments [8]. We highlight the contraction from a self-inflicted injection in a non-aquatic environment. After the introduction from injection, our patient developed a polymicrobial gaseous and necrotic soft tissue infection in the right hip that required debridement. 

The susceptibility tests of L. adecarboxylata cultured from our patient demonstrated resistance to Ampicillin. There have been reports of this organism developing New Delhi metallo-B-lactamase-1 (NDM-1) resistance, resulting in the ability to hydrolyze all B-lactams [9]. Although this patient was susceptible to other B-Lactam antibiotics, the development of Ampicillin resistance alone in an organism of growing prevalence leads to concern of future antibiotic resistance. The growth of L. adecarboxylata from culture drew concern for unregulated supplements as a new route for infection in immunocompetent hosts. 

Select athletes elect to use performance-enhancing drugs which can either come from professional or personal laboratories, with the purity and contamination differing significantly. Although it is not possible to differentiate the quality of a product by its packaging, liquid chromatography and tandem mass spectrometry provide valuable insight. 25% of confiscated German black market drugs, including steroids, revealed impure or completely wrong components [10]. Many clandestine drug production operations have also been linked to integrating bacteria-contaminated water in their products [11]. Some users who obtained anabolic steroids from an illicit source were also more likely to engage in high-risk injection practices such as sharing multi-dose vials and dividing drugs using syringes [12]. These practices increase the risk of infection with organisms not classically associated with skin flora [13]. This is a possible explanation for our patient’s presentation with L. adecarboxylata infection following injections.

## Conclusions

This case report highlights the case of an immunocompetent individual contracting L. adecarboxylata as part of a polymicrobial gaseous and necrotizing soft tissue infection in a non-traumatic and non-aquatic environment. Our patient was injecting non-sterile testosterone and herbal supplements into her right thigh which introduced these bacteria, eventually resulting in a debilitating tissue infection requiring surgical intervention. This is the first case, to our knowledge, which demonstrates an infection with L. adecarboxylata in an immunocompetent individual through this route. 

As our patient purchased these supplements from a street vendor, this case raises questions regarding the safety of an unregulated market. Especially considering the resistance to ampicillin, there is concern for growth of newly resistant organisms. We hope this case brings to attention this new avenue for infection with L. adecarboxylata in immunocompetent hosts and highlights the importance of screening for this organism. We believe that awareness regarding the clinical presentation and potential antibiotic resistance can assist in clinical care moving forward.
